# Supplementary material for: Unveiling the A-to-I mRNA editing machinery and its regulation and evolution in fungi
Source: Nat Commun. 2024 May 10;15:3934. doi: 10.1038/s41467-024-48336-8 (PMC11087585; doi:10.1038/s41467-024-48336-8)
Supplement: Supplementary file 1 — Supplementary Information [file 41467_2024_48336_MOESM1_ESM.pdf]

Supplementary Information

Unveiling the A-to-I mRNA editing machinery and its regulation and evolution in fungi

Chanjing Feng[1], Kaiyun Xin[1], Yanfei Du[1], Jingwen Zou[1], Xiaoxing Xing[1], Qi Xiu[1], Yijie Zhang[1], Rui Zhang[2], Weiwei Huang[2], Qinhu Wang[1], Cong Jiang[1], Xiaojie Wang[1], Zhensheng Kang[1], Jin-Rong Xu[3], Huiquan Liu[1]*

[1] State Key Laboratory for Crop Stress Resistance and High-Efficiency Production, College of Plant Protection, Northwest A&F University, Yangling, Shaanxi 712100, China

[2] College of Life Sciences, Northwest A&F University, Yangling, Shaanxi 712100, China

[3] Department of Botany and Plant Pathology, Purdue University, West Lafayette, IN 47907, USA

* Corresponding author. Email: liuhuiquan@nwsuaf.edu.cn

This PDF file includes:

Supplementary Fig. 1 to Fig. 10

Supplementary Table 1

Uncropped scans of all blots for Supplementary Fig. 9

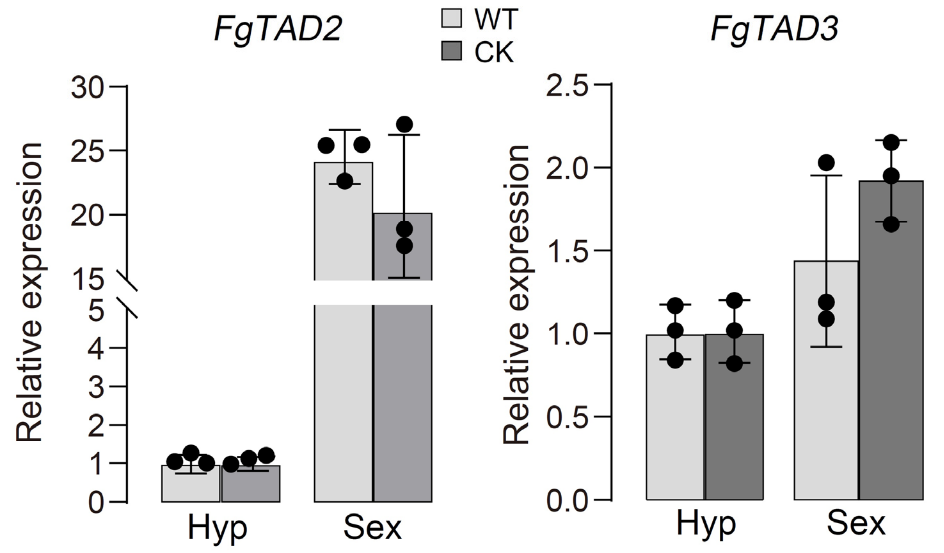

Supplementary Fig. 1 | Fold changes in the expression of *FgTAD2* and *FgTAD3* in perithecia (Sex) compared to hyphae (Hyp) of the wild type (WT) and control check (CK) strain assessed using qRT-PCR. The CK strain refers to the FG1G36140 deletion mutant utilized for the overexpression of *FgTAD3* and *FgTAD2* at the FG1G36140 locus. Data are presented as mean±SD, n = 3 independent replicates. Source data are provided as a Source Data file.

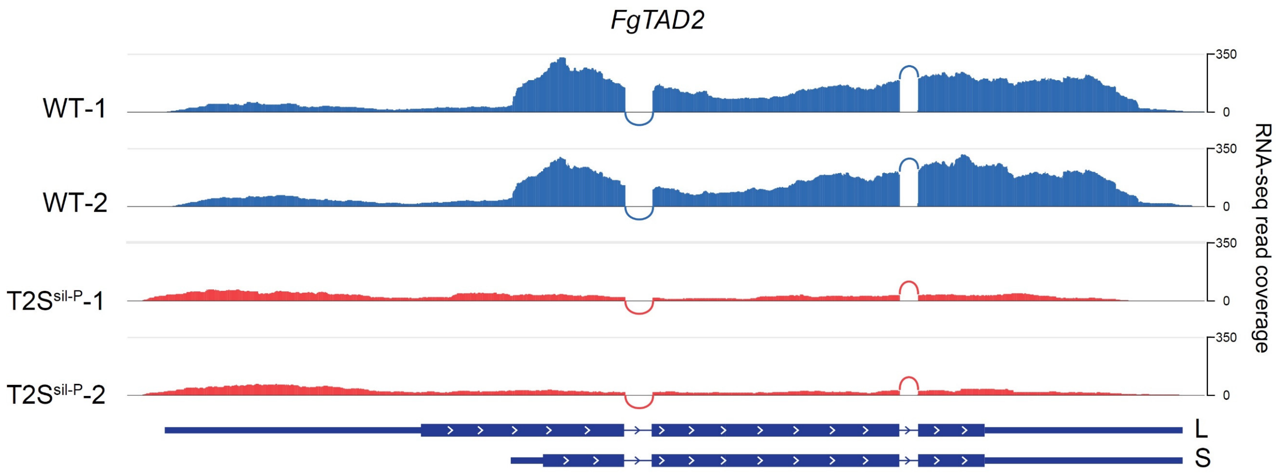

Supplementary Fig. 2 | RNA-seq read coverage showing the expression of the long (L) and short (S) transcripts of *FgTAD2* in 7-dpf perithecia of the wild type (WT) and T2S$^{sil\text{-}P}$ mutant. For each strain, data of two biological replicates are shown.

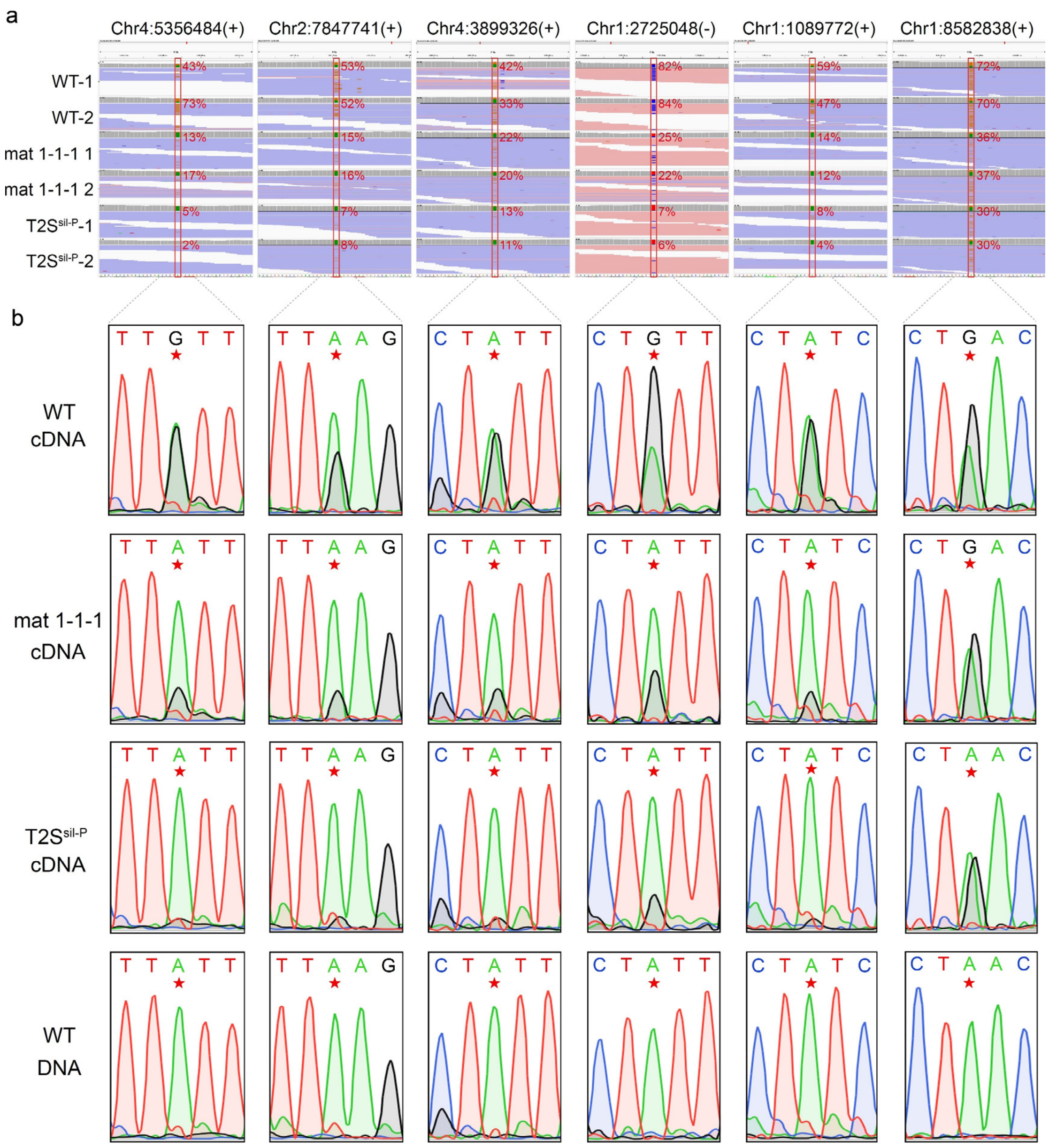

Supplementary Fig. 3 | Validation of A-to-I mRNA editing sites identified by RNA-seq using Sanger sequencing. a RNA-Seq read mapping showing six A-to-I mRNA editing sites detected in perithecia of the designated strains. Red boxes highlight the A-to-I mRNA editing sites, with their editing levels indicated. Data of two biological replicates (-1 and -2) are shown. **b** Validation of the A-to-I mRNA editing sites through Sanger sequencing of the RT-PCR (cDNA) and PCR (gDNA) products. The editing sites are marked by a red star. When editing occurs, two peaks (A and G) at the editing sites are observed in the sequencing traces.

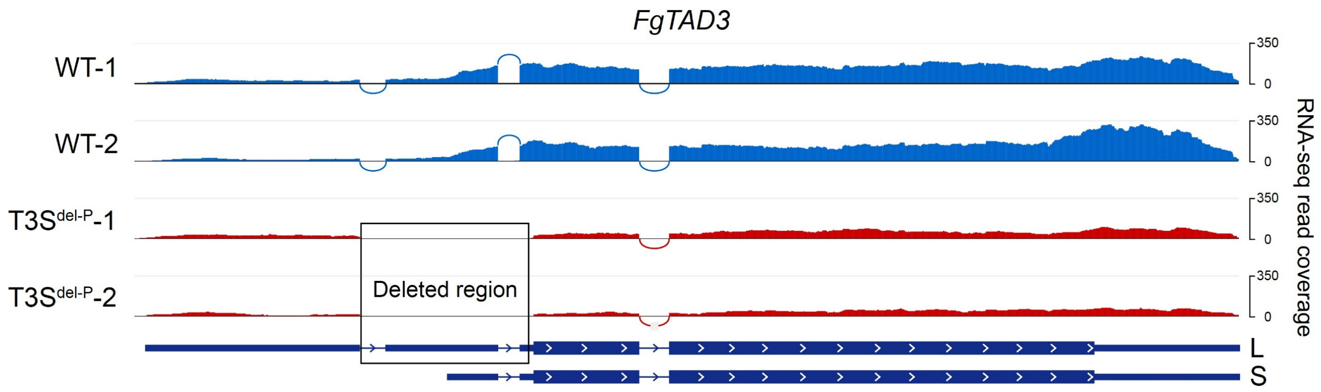

Supplementary Fig. 4 | RNA-seq read coverage showing the expression of the long (L) and short (S) transcripts of *FgTAD3* in 7-dpf perithecia of the wild type (WT) and T3S$^{del\text{-}P}$ mutant. For each strain, data of two biological replicates are shown. The deleted promoter region of the S-transcript in the T3S$^{del\text{-}P}$ mutant is indicated.

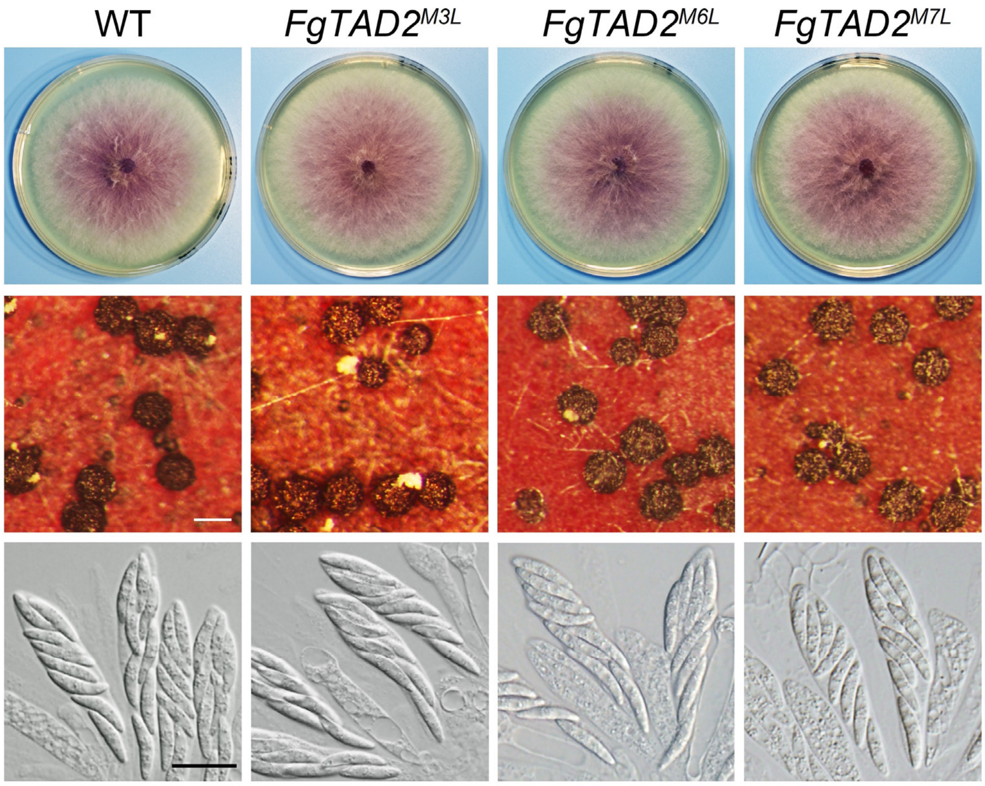

Supplementary Fig. 5 | Normal vegetative growth and sexual development in *FgTAD2*^{M3L}, *FgTAD2*^{M6L}, and *FgTAD2*^{M7L} mutants. White bar = 0.2 mm; black bar = 20 μm. All images shown are representative of consistent results from at least three independent experiments.

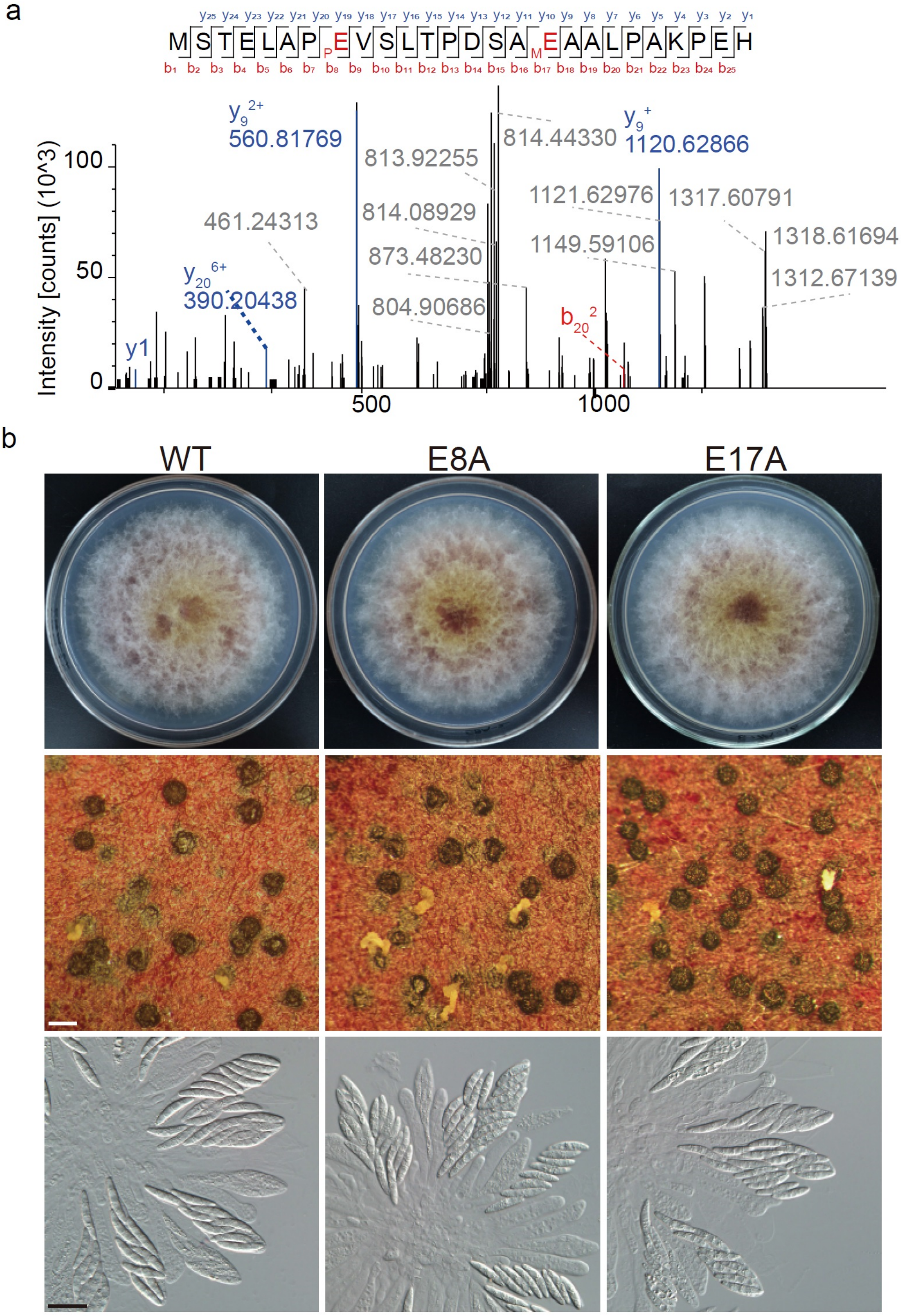

Supplementary Fig. 6 | Normal vegetative growth and sexual development in the post-translational modification-deficient mutants *FgTAD3*^{E8A} and *FgTAD3*^{E17A}. a MS/MS spectrum of the peptide containing the phosphorylated (pE^8) and methylated (mE^{17}) residues. **b** Colony morphology, perithecium formation, and asci/ascospores morphology of the modification-deficient mutants *FgTAD3*^{E8A} and *FgTAD3*^{E17A}. White bar = 0.2 mm; black bar = 20 μm. All images shown are representative of consistent results from at least three independent experiments.

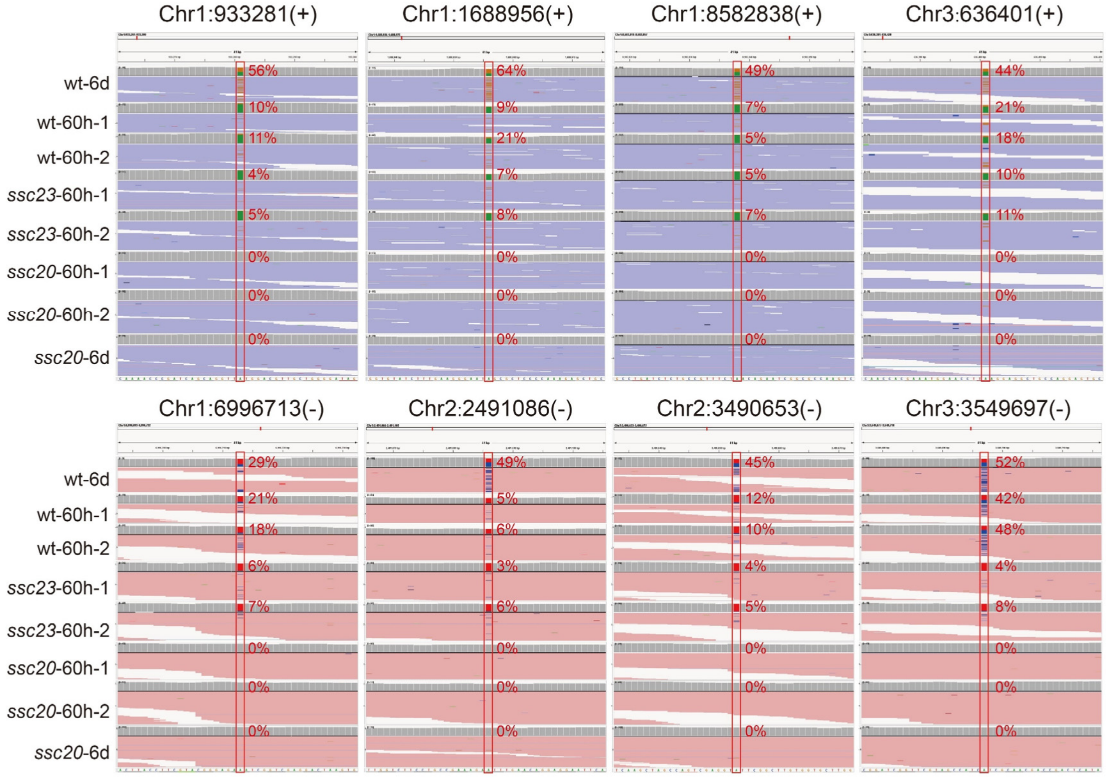

Supplementary Fig. 7 | IGV read mapping showing four A-to-I mRNA editing sites detected in RNA-Seq data of the wild type (wt) and *ssc23* deletion mutant but not the *ssc20/ame1* deletion mutant. Red boxes highlight the A-to-I mRNA editing sites, with their editing levels indicated. Data of two biological replicates (-1 and -2) are shown.

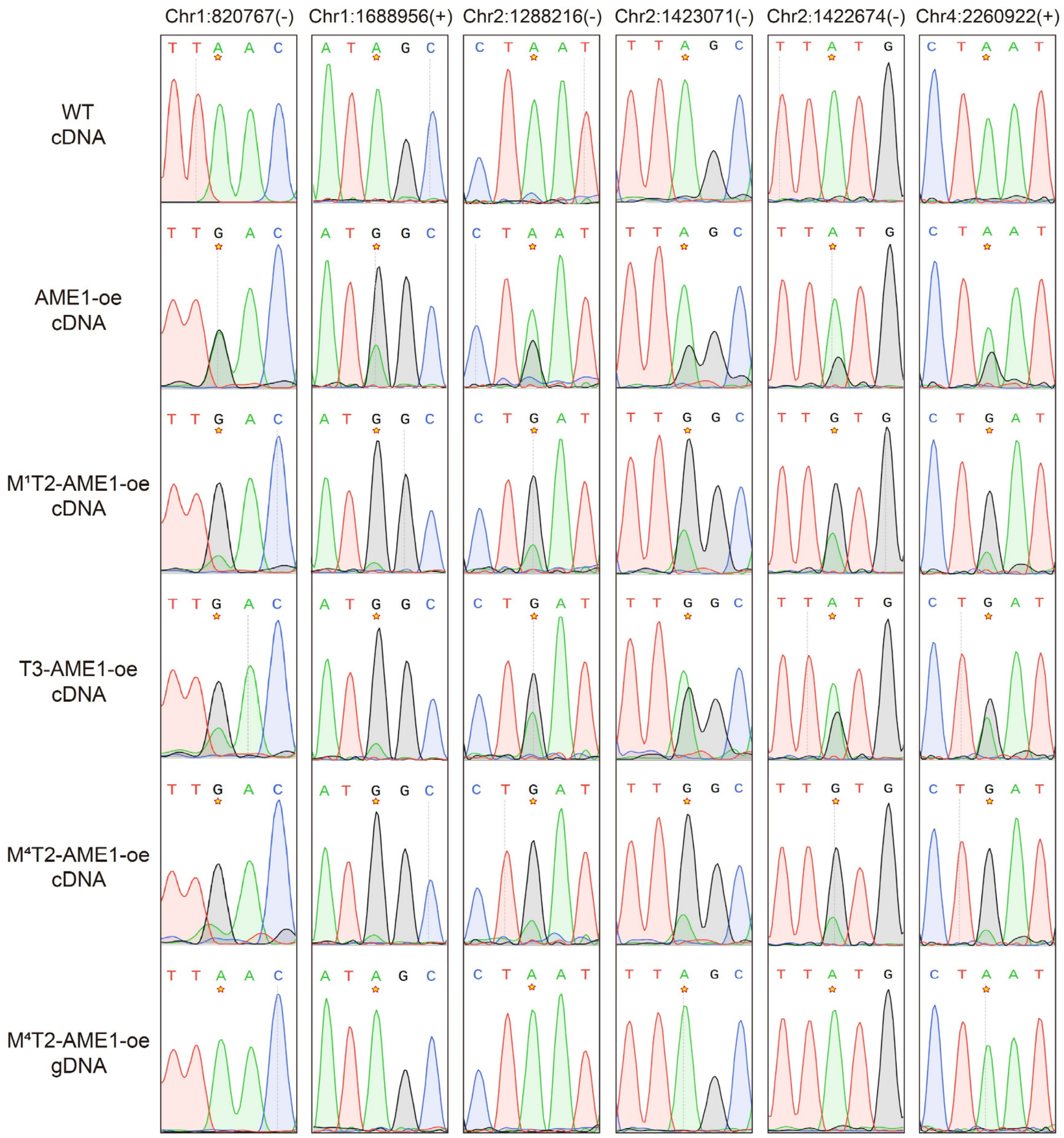

Supplementary Fig. 8 | Validation of A-to-I mRNA editing sites identified in 24-h hyphae of the designated strains by RNA-seq using Sanger sequencing of the RT-PCR (cDNA) and PCR (gDNA) products. The editing sites are marked by a red star. When editing occurs, two peaks (A and G) at the editing sites are observed in the sequencing traces.

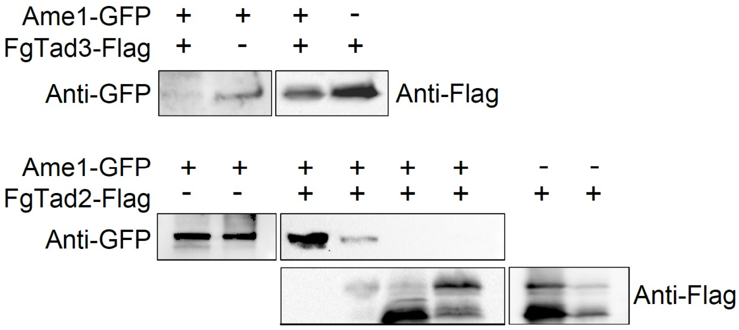

Supplementary Fig. 9 | Western blots showing the expression of Ame1-GFP suppressed when co-expressed with FgTad2-FLAG or FgTad3-FLAG. All images shown are representative of consistent results from at least three independent experiments.

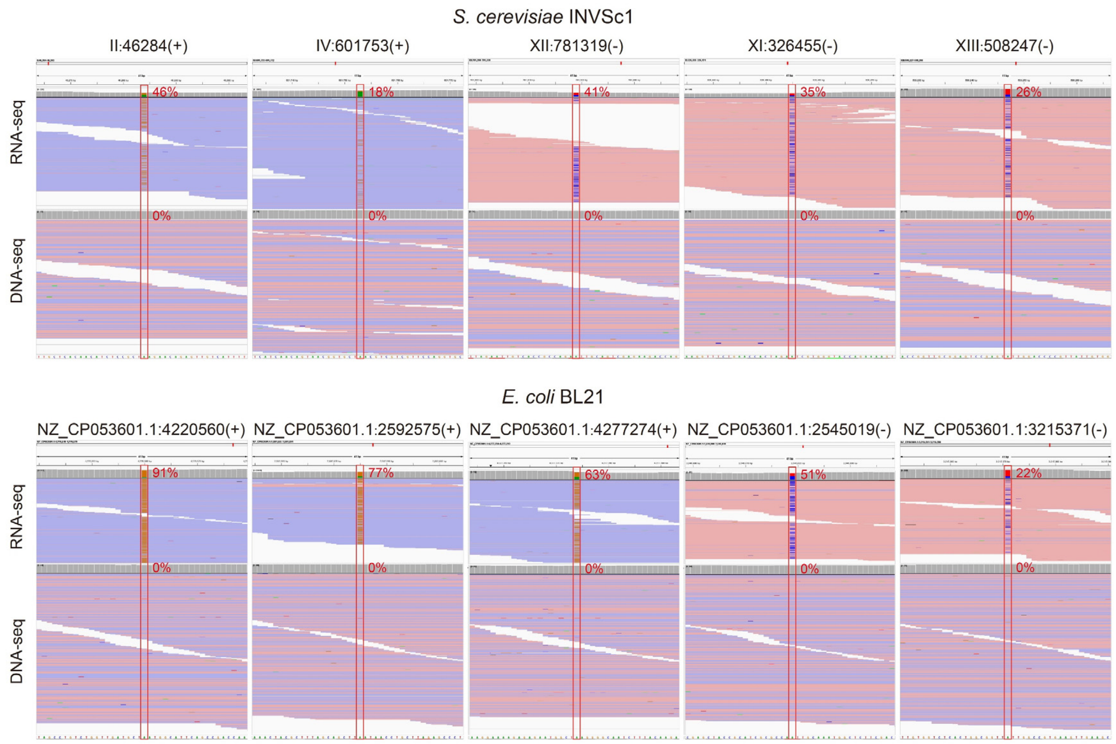

Supplementary Fig. 10 | IGV read mapping showing A-to-I mRNA editing events detected in RNA-Seq data but not DNA-seq data of the yeast INvSC1 strain and *E. coli* BL21 strain expressing the FgTad2-FgTad3-Ame1 complex. Red boxes highlight the A-to-I mRNA editing sites, with their editing levels indicated.

Supplementary Table 1 | Number of A-to-I RNA editing sites detected in each strain.

Type	WT-60h-1	WT-60h-2	*ssc23*-60h-1	*ssc23*-60h-2	*ssc20*-60h-1	*ssc20*-60h-2	*ssc20*-6d	*FgTAD3*^{M120I}-7d	WT-Hy24h-1	WT-Hy24h-2
AC	22	23	25	29	34	42	15	96	6	10
AG*	151 (141)	171 (162)	34 (23)	40 (28)	14 (0)	15 (0)	16 (0)	52 (40)	12 (0)	15 (1)
AT	17	14	23	15	18	20	17	33	20	20
CA	13	16	18	12	11	20	10	53	17	8
CG	9	9	9	9	8	10	7	14	9	9
CT	15	15	14	21	21	21	23	60	42	31
GA	80	79	87	64	77	99	82	53	29	26
GC	8	12	14	9	9	14	12	13	12	9
GT	29	29	35	24	37	26	38	86	52	54
TA	15	15	15	13	13	18	18	21	21	24
TC	38	45	47	53	44	63	49	47	35	27
TG	26	19	23	32	19	21	12	44	14	10

*The number in parentheses represents the count of A-to-I RNA editing sites that have been manually confirmed using IGV and RNA-seq BAM files. Data of two biological replicates (-1 and -2) are shown.

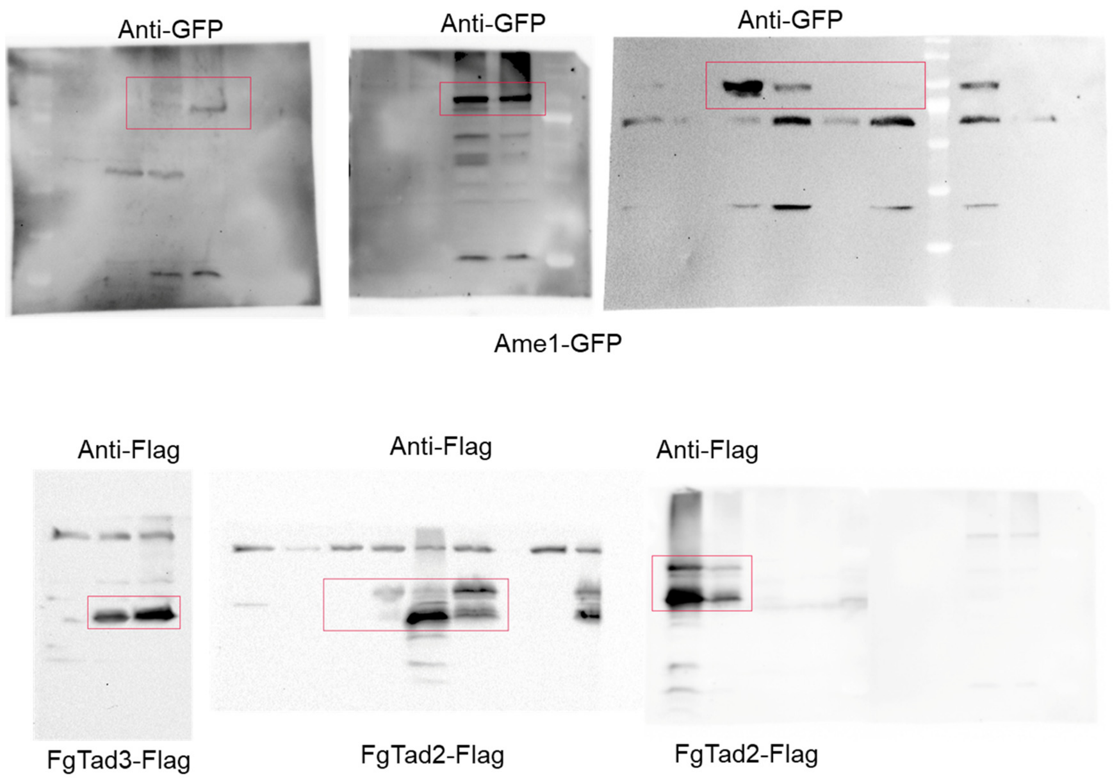

Uncropped scans of all blots for Supplementary Fig. 9
