# Supplementary material for: Unveiling the A-to-I mRNA editing machinery and its regulation and evolution in fungi
Source: Nat Commun. 2024 May 10;15:3934. doi: 10.1038/s41467-024-48336-8 (PMC11087585; doi:10.1038/s41467-024-48336-8)
Supplement: Supplementary file 3 — Description of Additional Supplementary Files [file 41467_2024_48336_MOESM3_ESM.pdf]

Description of Additional Supplementary Files

File Name: Supplementary Data 1
Description: Information of 34 sexual stage-specific conserved (SSC) genes.

File Name: Supplementary Data 2
Description: Amino acid mutations of FgTad3 and defective phenotypes in the ascospore progeny of the TR-FgTAD3 strain.

File Name: Supplementary Data 3
Description: Strains used in this study.

File Name: Supplementary Data 4
Description: Primers used in this study.

File Name: Supplementary Data 5
Description: Information of Illumina DNA- and RNA-Seq data used in this study.

File Name: Supplementary Data 6
Description: Information of Ame1 orthologs used for phylogeny.
