# Supplementary material for: Unveiling the A-to-I mRNA editing machinery and its regulation and evolution in fungi
Source: Nat Commun. 2024 May 10;15:3934. doi: 10.1038/s41467-024-48336-8 (PMC11087585; doi:10.1038/s41467-024-48336-8)
Supplement: Supplementary file 10 — Reporting Summary [file 41467_2024_48336_MOESM10_ESM.pdf]

nature portfolio

Corresponding author(s): Huiquan Liu

Last updated by author(s): Apr 19, 2024

Reporting Summary

Nature Portfolio wishes to improve the reproducibility of the work that we publish. This form provides structure for consistency and transparency in reporting. For further information on Nature Portfolio policies, see our Editorial Policies and the Editorial Policy Checklist.

Statistics

For all statistical analyses, confirm that the following items are present in the figure legend, table legend, main text, or Methods section.

n/a	Confirmed	
☐	☒	The exact sample size (*n*) for each experimental group/condition, given as a discrete number and unit of measurement
☐	☒	A statement on whether measurements were taken from distinct samples or whether the same sample was measured repeatedly
☐	☒	The statistical test(s) used AND whether they are one- or two-sided<br>*Only common tests should be described solely by name; describe more complex techniques in the Methods section.*
☒	☐	A description of all covariates tested
☐	☒	A description of any assumptions or corrections, such as tests of normality and adjustment for multiple comparisons
☐	☒	A full description of the statistical parameters including central tendency (e.g. means) or other basic estimates (e.g. regression coefficient) AND variation (e.g. standard deviation) or associated estimates of uncertainty (e.g. confidence intervals)
☒	☐	For null hypothesis testing, the test statistic (e.g. *F*, *t*, *r*) with confidence intervals, effect sizes, degrees of freedom and *P* value noted<br>*Give P values as exact values whenever suitable.*
☒	☐	For Bayesian analysis, information on the choice of priors and Markov chain Monte Carlo settings
☒	☐	For hierarchical and complex designs, identification of the appropriate level for tests and full reporting of outcomes
☒	☐	Estimates of effect sizes (e.g. Cohen's *d*, Pearson's *r*), indicating how they were calculated

Our web collection on statistics for biologists contains articles on many of the points above.

Software and code

Policy information about availability of computer code

Data collection	No software was used.
Data analysis	AlphaFold-Multimer (http://cosmic-cryoem.org/) for protein structure modeling.<br>BamTools v2.5.2 (https://hcc.unl.edu/docs/applications/app_specific/bioinformatics_tools/data_manipulation_tools/bamtools/) for separating sense-strand and antisense-strand read alignments.<br>BLASTp (https://blast.ncbi.nlm.nih.gov/Blast.cgi) for homologous sequence detection.<br>Bowtie 2 v2.5.1 (https://bowtie-bio.sourceforge.net/bowtie2/index.shtml) for DNA-seq reads mapping.<br>ChimeraX v1.4 (https://www.cgl.ucsf.edu/chimerax/) for molecular visualization.<br>featureCounts v1.5.1 (https://subread.sourceforge.net/featureCounts.html) for read summarization.<br>forgi v2.0.3 (https://github.com/ViennaRNA/forgi/releases) for statistics of secondary structure types.<br>HISAT 2.0.4 (http://daehwankimlab.github.io/hisat2/) for RNA-seq reads mapping.<br>IQ-TREE v2.2.2.7 (http://www.iqtree.org/) for phylogeny analysis.<br>MUSCLE v5.1 (https://www.drive5.com/muscle/) for multiple sequence alignments.<br>NCBI CD-Search (https://www.ncbi.nlm.nih.gov/Structure/cdd/wrpsb.cgi) for conserved domain searches.<br>orthAgogue (https://code.google.com/archive/p/orthagogue/wikis/Manual.wiki) for ortholog identification.<br>Picard package v2.18.7 (http://broadinstitute.github.io/picard/) for removing duplicated reads in the mapped RNA-seq BAM file.<br>PRIME v2.0.1 (https://www.schrodinger.com/products/prime) for molecular docking.<br>Qualimap v2.2.1 (http://qualimap.conesalab.org/) for the quality control of alignment sequencing data.<br>REDItools v1.2 (https://github.com/BioinfoUNIBA/REDItools) for A-to-I RNA editing identification.<br>Trimmomatic v0.39 (https://github.com/usadellab/Trimmomatic) for removing low-quality reads and reads containing adapters.

Viennarna v2.4.18 (https://github.com/ViennaRNA/ViennaRNA/releases) for RNA secondary structure predication.

For manuscripts utilizing custom algorithms or software that are central to the research but not yet described in published literature, software must be made available to editors and reviewers. We strongly encourage code deposition in a community repository (e.g. GitHub). See the Nature Portfolio guidelines for submitting code & software for further information.

Data

Policy information about availability of data

All manuscripts must include a data availability statement. This statement should provide the following information, where applicable:

- Accession codes, unique identifiers, or web links for publicly available datasets
- A description of any restrictions on data availability
- For clinical datasets or third party data, please ensure that the statement adheres to our policy

Data supporting the findings of this work are available within the paper and its Supplementary Information file. Source data are provided with this paper. Omics data generated in this study are accessible under the NCBI BioProject accession: PRJNA1020378 [https://www.ncbi.nlm.nih.gov/bioproject/PRJNA1020378/]. Published omics data used in this study are accessible under the NCBI BioProject accession: PRJNA384311 [https://www.ncbi.nlm.nih.gov/bioproject/PRJNA384311] and SRA accessions: SRR3030980 [https://www.ncbi.nlm.nih.gov/sra/?term=SRR3030980], SRR2182495 [https://www.ncbi.nlm.nih.gov/sra/?term=SRR2182495], SRR2182497 [https://www.ncbi.nlm.nih.gov/sra/?term=SRR2182497], SRR2182499 [https://www.ncbi.nlm.nih.gov/sra/?term=SRR2182499], and SRR2182501[https://www.ncbi.nlm.nih.gov/sra/?term=SRR2182501].

Research involving human participants, their data, or biological material

Policy information about studies with human participants or human data. See also policy information about sex, gender (identity/presentation), and sexual orientation and race, ethnicity and racism.

Reporting on sex and gender	*Use the terms sex (biological attribute) and gender (shaped by social and cultural circumstances) carefully in order to avoid confusing both terms. Indicate if findings apply to only one sex or gender; describe whether sex and gender were considered in study design; whether sex and/or gender was determined based on self-reporting or assigned and methods used. Provide in the source data disaggregated sex and gender data, where this information has been collected, and if consent has been obtained for sharing of individual-level data; provide overall numbers in this Reporting Summary. Please state if this information has not been collected. Report sex- and gender-based analyses where performed, justify reasons for lack of sex- and gender-based analysis.*
Reporting on race, ethnicity, or other socially relevant groupings	*Please specify the socially constructed or socially relevant categorization variable(s) used in your manuscript and explain why they were used. Please note that such variables should not be used as proxies for other socially constructed/relevant variables (for example, race or ethnicity should not be used as a proxy for socioeconomic status). Provide clear definitions of the relevant terms used, how they were provided (by the participants/respondents, the researchers, or third parties), and the method(s) used to classify people into the different categories (e.g. self-report, census or administrative data, social media data, etc.) Please provide details about how you controlled for confounding variables in your analyses.*
Population characteristics	*Describe the covariate-relevant population characteristics of the human research participants (e.g. age, genotypic information, past and current diagnosis and treatment categories). If you filled out the behavioural & social sciences study design questions and have nothing to add here, write "See above."*
Recruitment	*Describe how participants were recruited. Outline any potential self-selection bias or other biases that may be present and how these are likely to impact results.*
Ethics oversight	*Identify the organization(s) that approved the study protocol.*

Note that full information on the approval of the study protocol must also be provided in the manuscript.

Field-specific reporting

Please select the one below that is the best fit for your research. If you are not sure, read the appropriate sections before making your selection.

☒ Life sciences ☐ Behavioural & social sciences ☐ Ecological, evolutionary & environmental sciences

For a reference copy of the document with all sections, see nature.com/documents/nr-reporting-summary-flat.pdf

Life sciences study design

All studies must disclose on these points even when the disclosure is negative.

Sample size	No statistical method was used to predetermine sample size.
Data exclusions	No data were excluded from the analyses.
Replication	All replication attempts were successful. For RIP-seq, the results were presented for two independent experiments. For RNA-seq, in most

Replication	cases, two biological replicates were used. For phenotype observation, the images shown are representative of consistent results from three independent experiments with at least three biological replicates each. All the blot images presented represent consistent results from at least two independent experiments.
Randomization	The experiments were not randomized.
Blinding	The Investigators were not blinded to allocation during experiments and outcome assessment.

Reporting for specific materials, systems and methods

We require information from authors about some types of materials, experimental systems and methods used in many studies. Here, indicate whether each material, system or method listed is relevant to your study. If you are not sure if a list item applies to your research, read the appropriate section before selecting a response.

Materials & experimental systems

n/a	Involved in the study
☐	☒ Antibodies
☐	☒ Eukaryotic cell lines
☒	☐ Palaeontology and archaeology
☒	☐ Animals and other organisms
☒	☐ Clinical data
☒	☐ Dual use research of concern
☒	☐ Plants

Methods

n/a	Involved in the study
☒	☐ ChIP-seq
☒	☐ Flow cytometry
☒	☐ MRI-based neuroimaging

Antibodies

Antibodies used	anti-FLAG (Sigma-Aldrich, F31651), anti-GFP (Abcam, ab290), anti-His (CW0286M, CMBIO, China), anti-Stag (Cell Signaling Technology, 12774S), anti-GAPDH (Sangon Biotech, D1100160200)
Validation	data provided in the manuscript.

Eukaryotic cell lines

Policy information about cell lines and Sex and Gender in Research

Cell line source(s)	The used HEK293 cell line (Servicebio, China) consists of immortalized human embryonic kidney cells is one of the most widely used cell lines in research.
Authentication	Cell lines were verified by examination of cell morphology under microscope and genotyping by PCR.
Mycoplasma contamination	The cell lines used were test for Mycoplasma contamination using a Mycoplasma Detection Kit (InvioGen).
Commonly misidentified lines (See ICLAC register)	None of commonly misidentified lines were used in this study.
